# Dengue Dynamics and Vaccine Cost-Effectiveness Analysis in the Philippines

**DOI:** 10.4269/ajtmh.16-0194

**Published:** 2016-11-02

**Authors:** Eunha Shim

**Affiliations:** 1 Department of Mathematics, Soongsil University, Seoul, Republic of Korea

## Abstract

Dengue is one of the most problematic vector-borne diseases in the Philippines, with an estimated 842,867 cases resulting in medical costs of $345 million U.S. dollars annually. In December 2015, the first dengue vaccine, known as chimeric yellow fever virus–dengue virus tetravalent dengue vaccine, was approved for use in the Philippines and is given to children 9 years of age. To estimate the cost-effectiveness of dengue vaccination in the Philippines, we developed an age-structured model of dengue transmission and vaccination. Using our model, we compared two vaccination scenarios entailing routine vaccination programs both with and without catch-up vaccination. Our results indicate that the higher the cost of vaccination, the less cost-effective the dengue vaccination program. With the current dengue vaccination program that vaccinates children 9 years of age, dengue vaccination is cost-effective for vaccination costs up to $70 from a health-care perspective and up to $75 from a societal perspective. Under a favorable scenario consisting of 1 year of catch-up vaccinations that target children 9–15 years of age, followed by regular vaccination of 9-year-old children, vaccination is cost-effective at costs up to $72 from a health-care perspective and up to $78 from a societal perspective. In general, dengue vaccination is expected to reduce the incidence of both dengue fever and dengue hemorrhagic fever /dengue shock syndrome. Our results demonstrate that even at relatively low vaccine efficacies, age-targeted vaccination may still be cost-effective provided the vaccination cost is sufficiently low.

## Introduction

Dengue is the leading cause of vector-borne viral disease in humans, resulting in 390 million infections and 96 million symptomatic cases annually worldwide.^1^ Dengue infection poses a heavy economic burden to the health system in a society. The region with the highest dengue incidence is southeast Asia (SEA), where cycles of epidemics occur every 3–5 years.^2^ The SEA and the Western Pacific represent about 75% of the current global burden of dengue, and the Philippines is among the most affected.^2^ In the Philippines, the first epidemic of severe dengue was documented in Manila in 1953, and since then, dengue has been hyperendemic in most areas of the country with an increasing number of dengue cases over time.^1^ With an adjustment for underreported dengue cases, a recent study estimated an annual average of 842,867 clinically diagnosed cases of dengue in the Philippines, with direct medical costs of $345 million U.S. dollars.^3^

Dengue fever (DF) is caused by one of the four distinct serotypes of dengue virus (DENV), DENV 1–4. Infection with one serotype provides life-long immunity against reinfection with that particular serotype, but not against the others. The first infection is normally asymptomatic or presents only mild symptoms. However, severe diseases, including dengue hemorrhagic fever (DHF) and dengue shock syndrome (DSS), mostly occur among individuals who have already recovered from the first infection and are experiencing a secondary infection with a different serotype.^4^

Vaccination is considered one of the most cost-effective prevention strategies to lower the burden of dengue, particularly in children, in both developing and developed countries.^5^ The World Health Organization (WHO) has called for the development of a dengue vaccine as an essential part of the integrated dengue prevention effort needed to lower the dengue burden and dengue-related fatalities globally before 2020.

On December 23, 2015, the Philippines became the first country in Asia to license the world's first dengue vaccine, a live recombinant chimeric yellow fever virus–DENV tetravalent dengue vaccine (CYD-TDV) called Dengvaxia.^6^ This vaccine has been approved in Mexico and Brazil^6^,^7^ for use in individuals 9–45 years of age living in endemic areas. It will be administrated in three doses with a 6-month interval between each dose. Results from the phase III randomized, controlled vaccine trials of CYD-TDV reported a relatively low vaccine efficacy of 57% against virologically confirmed dengue.^8^ In the Philippines, the Department of Health launched the dengue school-based immunization program to give the dengue vaccines to Grade 4 public school students 9 years of age.

However, to date, few published studies have examined the economic and disease burden of dengue in the Philippines.^2^,^3^ Although prior cost-effectiveness analyses of dengue vaccination provided valuable results, a substantial amount of additional information has emerged recently, including vaccine safety, efficacy, and target ages. Thus, prior cost-effectiveness analyses have not assessed the new school-based program of dengue vaccination targeting individuals 9 years of age.

Our study is the first to assess cost-effectiveness of dengue vaccination in the Philippines. We estimated the economic and epidemiological impact of dengue vaccination in the Philippines and calculated its cost-effectiveness at various vaccine costs with and without a catch-up dengue vaccination program. Specifically, we developed an age-structured, dynamic dengue transmission model and used it to estimate the cost-effectiveness of the dengue vaccine in the Philippines, which allowed us to identify a threshold vaccine cost at which the dengue vaccine becomes cost-effective.

## Methods

### Mathematical model of dengue transmission and vaccination.

**Figure 1.**
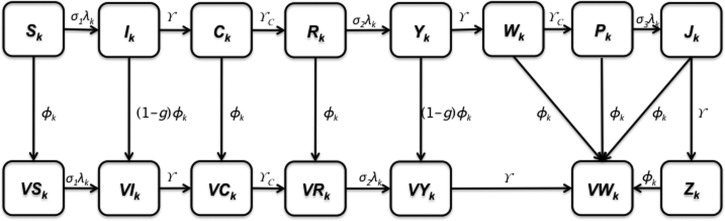
Model diagram. The population is divided into dengue-related age-dependent epidemiological classes. The subscript *k* indicates the age groups (*k* = 1, …, 15).

We constructed a deterministic, age-structured, compartmental model that captures key features of dengue transmission: clinical cross-immunity among the multiple serotypes of dengue, population age structure, and age-specific levels of transmission (Figure 1

). Our model includes primary, secondary, and tertiary infections. Two prior dengue infections are known to provide protective immunity against severe dengue disease accompanying infection with a third dengue infection.^9^,^10^ Therefore, third infections from dengue are likely to be asymptomatic, consistent with our model assumptions.^9^,^11^–^13^ We account for antibody-dependent enhancement by assuming that the probability of developing DHF and DSS after a secondary infection is greater than that after a primary infection.^14^,^15^

In our model, the population contains 15 distinct age classes, which represent individuals ≥ 0–4, 5–8, 9, 10–14, 15–19, 20–25, …, 60–64, and 65 years of age. Transition rates among these age classes are independent of infection status and occur through aging at rate *p*_*k*_ (*k* = 1, …, 15), where *p*_15_ = 0. Here, the subscript *k* refers to the age group *k*. Within each age class, we incorporate susceptible unvaccinated individuals (*S*_*k*_), primarily infected unvaccinated individuals (*I*_*k*_), unvaccinated individuals recovering from primary infections who are temporarily protected against clinical disease (*C*_*k*_), unvaccinated individuals susceptible to secondary infections (*R*_*k*_), unvaccinated individuals with secondary infections (*Y*_*k*_), unvaccinated individuals recovering from secondary infections (*W*_*k*_), unvaccinated individuals recovering from secondary infections who are temporarily protected against clinical disease (*P*_*k*_), unvaccinated individuals with tertiary infections (*J*_*k*_), unvaccinated individuals recovering from tertiary infections (*Z*_*k*_), partially susceptible vaccinated individuals (*V*_*k*_), primarily infected vaccinated individuals (*VI*_*k*_), vaccinated individuals recovering from primary infections and temporarily protected against clinical disease (*VC*_*k*_), vaccinated individuals susceptible to secondary infections (*VR*_*k*_), vaccinated individuals with secondary infections (*VY*_*k*_), and vaccinated individuals recovering from secondary infections (*VW*_*k*_) (Figure 1). The proportion, *g*, of infected individuals is assumed to be symptomatic. Unvaccinated individuals who recover from third infections (*Z*_*k*_) or vaccinated individuals who recover from secondary infections (*VW*_*k*_) are assumed to be immune to all strains. The rates of birth and death are denoted by *b* and μ_*k*_, respectively.

To capture the patterns of age-dependent incidence rates in the Philippines, our model considers age-dependent infection rates. Specifically, we define β_*k*_ as the age-dependent transmission rate among age group *k*. Our model combines the underlying process of vector contact with humans, and the dynamics of infection in the vector and subsequent transmission to other humans into one aggregate rate.^14^–^28^ Such an aggregate rate, denoted by β_*k*_ in our model, represents the mean vector-mediated rate at which humans infect other humans. Therefore, instead of considering separate contact rates for transmission from humans to vectors and vice versa, our model considers the rate of infection of susceptibles in age group *k* (i.e., the force of infection, λ_*k*_) where 
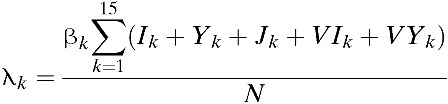
. That is, the force of infection (λ_*k*_) is assumed to be regulated by the number of infectious individuals and the transmission coefficient (β_*k*_). Infected individuals are assumed to recover from primary infections at rate γ and gain clinical cross-protection, which prevents clinical illness but allows seroconversion. The average duration of clinical cross-protection is assumed to be 1/γ_*C*_ (Table 1).

**Figure 2.**
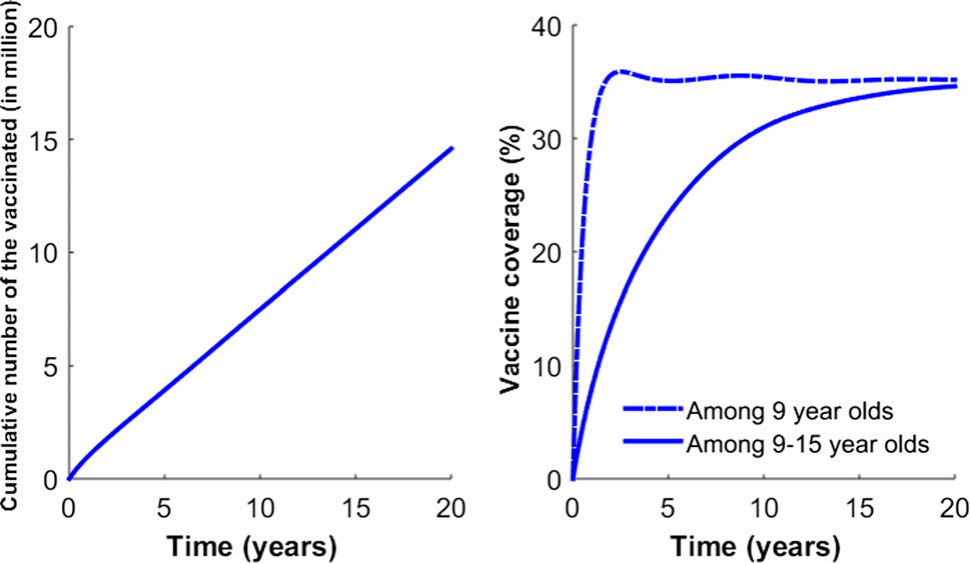
Vaccination coverage levels based on Strategy A. For Strategy A, vaccines are given to individuals 9 years of age. (**A**) The number of cumulative number of vaccinated individuals is presented. (**B**) The vaccination coverage level based on Strategy A is presented.

Our vaccination strategy is implemented by vaccinating individuals 9 years of age, consistent with current recommendations for the administration of the dengue vaccine in the Philippines. Specifically, for age group *k* = 3, individuals except those who are symptomatically infected are vaccinated at the rate of ϕ_*k*_ (Figures 2

and 3

**Figure 3.**
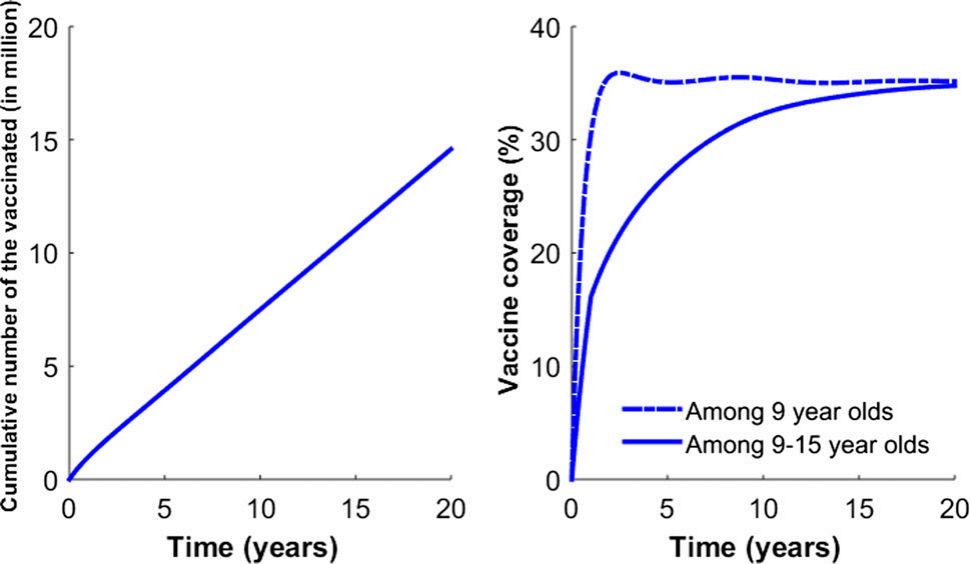
Vaccination coverage levels based on Strategy B. Strategy B consists of 1 year of catch-up targeting children 9–15 years of age, followed by regular vaccination of 9-year-old individuals. (**A**) The number of cumulative number of vaccinated individuals is presented. (**B**) The vaccination coverage level based on Strategy B is presented.

). To evaluate the potential impact of catch-up vaccination, we also considered vaccinating individuals 9–15 years of age when incorporating the catch-up vaccination program in addition to the regular vaccination of 9-year-old individuals.

The estimated efficacy from dengue vaccine trials has been expressed in terms of reduction of clinically apparent infection, which is distinct from vaccine efficacy against infection.^36^ Therefore, we assumed the vaccine efficacy against disease after infection, rather than the efficacy against infection, by incorporating the vaccine trials data into the model. Also, we assumed that the vaccine efficacy is dependent on the severity of infection and serological status, consistent with the vaccine trials data.^8^,^9^ Specifically, in our model, vaccine efficacy against both asymptomatic and symptomatic infection is denoted by ϵ and δ among individuals ≥ 9 years of age who had never been exposed to DENV (referred to as seronegative individuals) and individuals ≥ 9 years of age who had previously been exposed to DENV (referred to as seropositive individuals), respectively (ϵ < δ). The dengue vaccine trials data are only based on symptomatic infection, and thus, we assumed that the dengue vaccine efficacy against asymptomatic is the same as the vaccine efficacy against symptomatic infection.^8^,^9^ In addition, δ_*D*_ is defined as the vaccine efficacy against DHF among seropositive individuals ≥ 9 years of age.

Our model incorporates both vaccine-induced protection and vaccine-enhanced dengue disease among vaccine recipients, as observed in the CYD-TDV trials.^9^,^9^ Results of phase III efficacy trials of CYD-TDV conducted in Asia and Latin America demonstrated that an individual's age and DENV serostatus before vaccination affect vaccine efficacy.^37^ Specifically, vaccine efficacy was greater in seropositive individuals compared with seronegative individuals (ϵ < δ).^37^,^38^ Furthermore, prior exposure to DENV had an important role in the longer-term (hospital) safety observations.^37^ Specifically, vaccination may present immunological similarities to an attenuated subclinical primary infection, and thus vaccination of seronegative individuals potentially increases the risk of DHF during a subsequent wild-type infection.^37^ Thus, in our model, the probability of developing DHF/DSS after primary symptomatic infection among unvaccinated individuals was assumed to be lower than individuals who were seronegative when vaccinated (*h*_1_ < *h*_2,*k*_). Here, *h*_1_ and *h*_2, *k*_ are defined as the probability of developing DHF among symptomatically infected individuals in *I*_*k*_ and *VI*_*k*_, respectively (Table 1). Using these notations and assumptions, the age-structured model of dengue transmission and vaccination is given by:

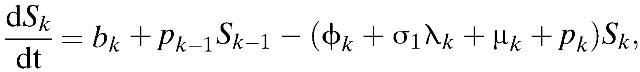


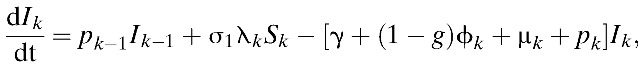


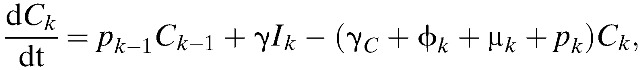


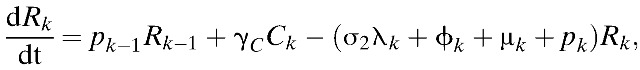


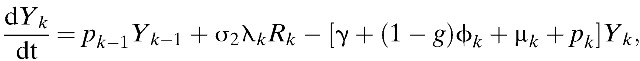


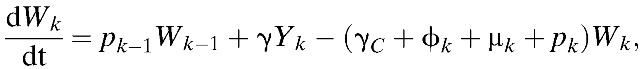


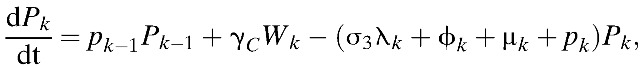


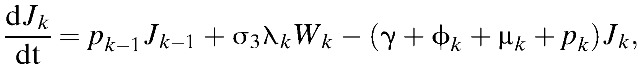


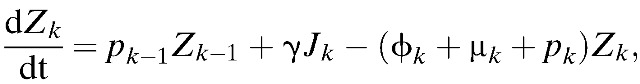


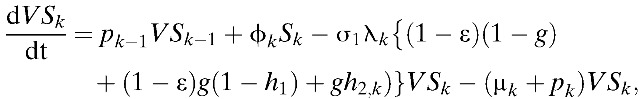


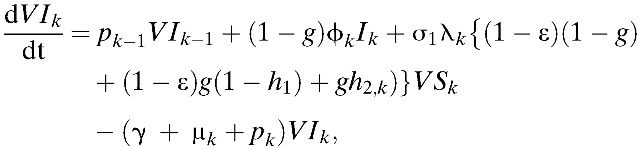


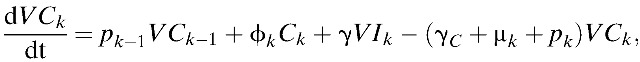


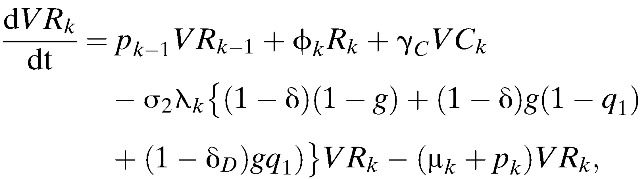


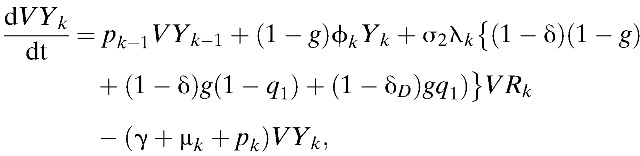


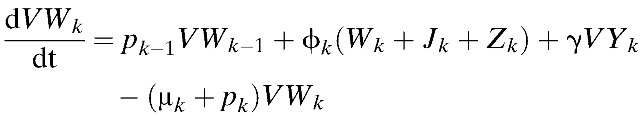


where 
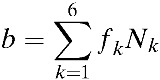
, 
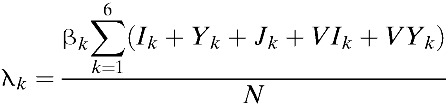
 and σ_*n*_ = (5 − *n*)/4. Here, σ_*n*_ is defined as a relative probability of being susceptible to *n*th infection. We complete the formulation by giving appropriate initial conditions: *S*_*k*_(0) = *S*_*k*,0_, *I*_*k*_(0) = *I*_*k*,0_, *C*_*k*_(0) = *C*_*k*,0_, *R*_*k*_(0) = *R*_*k*,0_, *Y*_*k*_(0) = *Y*_*k*,0_, *W*_*k*_(0) = *W*_*k*,0_, *P*_*k*_(0) = *P*_*k*,0_, *J*_*k*_(0) = *J*_*k*,0_, *Z*_*k*_(0) = *Z*_*k*,0_, and *VS*_*k*_(0) = *VI*_*k*_(0) = *VC*_*k*_(0) = *VR*_*k*_(0) = *VY*_*k*_(0) = *VW*_*k*_(0) = 0. Here, the initial conditions of the compartments within each age class differ due to immunity in older age groups. The relative size of *k*th age group is denoted by *N*_*k*_, where *N*_*k*_ = *S*_*k*_ + *I*_*k*_ + *C*_*k*_ + *R*_*k*_ + *Y*_*k*_ + *W*_*k*_ + *P*_*k*_ + *J*_*k*_ + *Z*_*k*_ + versus _*k*_ + *VI*_*k*_ + *VC*_*k*_ + *VR*_*k*_ + *VY*_*k*_ + *VW*_*k*_, 
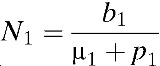
, 
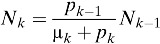
 for *k* = 2, …, 14, and 
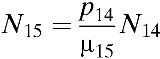
.

### Calibration.

**Figure 4.**
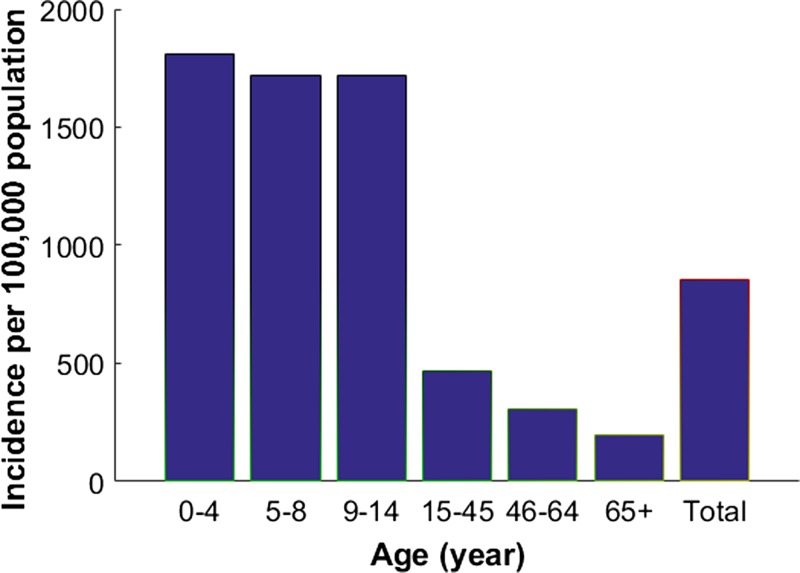
Annual number of symptomatic cases of dengue per 100,000 in age group in the prevaccine era. Reproduced from Bravo and others.^40^

Cases of dengue in the Philippines are known to be substantially underreported.^3^ The adjustment factor was estimated to be 7.2, meaning that for each reported case, there are 7.2 actual cases of dengue.^3^ Thus, we ran the model using baseline parameters and calibrated our model to an adjusted annual symptomatic dengue incidence of 0.84%, which incorporates underreported cases.^3^,^39^ This is comparable with the estimates of disease burden associated with dengue in other south Asian countries. For instance, in Thailand, its annual symptomatic dengue incidence, although underreported, ranges from 0.58% to 0.69%.^39^,^34^ In addition, 23% of primary and secondary dengue infections are assumed to be symptomatic.^32^,^33^ To generate country-specific dengue profiles for each age group, we allowed the transmission rates to be age dependent. These rates were chosen to capture the patterns of empirical dengue incidence in the Philippines (Figure 4

).^40^ Age-specific incidence profiles were obtained using β_1_ = 0.5121, β_2_ = 0.5536, β_3_ = 0.5536, β_4_ = 0.7058, β_5_ = β_6_ = β_7_ = β_8_ = β_9_ = β_10_ = 0.2768, β_11_ = β_12_ = β_13_ = β_14_ = 0.2007, and β_15_ = 0.1522. When incorporating adjustments to account for underreporting, the annual incidences of DHF in the Philippines is estimated to be 0.016%.^3^ These probabilities were varied for sensitivity analysis when we examined cost-effectiveness.

### Vaccination strategies.

Vaccination scenarios that model the impact of two different vaccination programs are presented (Figures 2 and 3). The first, called “Strategy A,” assumed that the rollout of the vaccine consisted of routine vaccination of 9-year-old individuals. Vaccination rates in routine programs were constant over time and set so that vaccination coverage would reach 1 million children after 1 year.^41^,^42^ These vaccination rates were chosen to roughly correspond with the rate of vaccination aimed for in the Philippines using a routine immunization campaign.^42^ “Strategy B” consisted of 1 year of catch-up targeting children 9–15 years of age, followed by regular vaccination of 9-year-old children. For Strategy B, the same vaccination rates in Strategy A were used in catch-up and routine programs.^43^

### Direct and indirect unit costs.

Our cost-effectiveness analysis was performed from both the health-care perspective (direct costs only) and the societal perspective (direct and indirect costs). In our analysis, all health and economic outcomes were discounted at a uniform rate of 3% per year, and all costs were standardized to 2016 U.S. dollars using the consumer price index.^44^ Direct medical costs of a treated hospitalized case averaged $869 in private hospitals and $437 in public hospitals, which results in a combined cost of $636 (Tables 2 and 3).^3^ For cases of dengue treated only in an ambulatory setting, the associated cost was $89 in the public sector and $189 in the private sector, which amounts to a combined cost of $135.^3^ Combining these cost estimates with the distribution of cases, we derived an average cost estimate per DF and DHF infection (i.e., *C*_DF, direct_ and *C*_DHF, direct_, respectively) (Table 3). The estimates of the indirect costs of hospitalized and ambulatory cases were obtained from prior studies based on bivariate regression.^2^ In this bivariate regression, Shepard and others extrapolated *ln*(indirect cost) as dependent variables, using *ln*(gross domestic product [GDP] per capita) as one of the independent variables.^2^ Specifically, indirect costs associated with hospitalized cases and ambulatory cases are estimated at $42 and $20 per individual, respectively. In addition, indirect costs in the Philippines associated with dengue-related deaths are estimated at $87,418 per death for children (< 15 years of age) and $56,822 per death for adults (≥ 15 years of age).^41^ We used the human capital approach to estimate the indirect cost of dengue deaths, by using the average ages of death and the average discounted life expectancy for children and adults based on WHO life tables.^41^,^53^ We estimated the discounted years of life lost for adults and children by first multiplying the number of fatal dengue episodes in each age group by the discounted years of life lost for that age group. The product was summed according to the age groups (i.e., child or adult), and we then computed the weighted average of the discounted years of life lost for each age group. The economic cost of each year of discounted life expectancy was valued at the GDP per capita in the Philippines. Finally, to estimate the indirect cost of fatal cases in children and in adults, the number of dengue fatal cases for children (or adults) was multiplied by the Philippines' GDP per capita and the corresponding discounted years of life lost.

### Calculation of quality-adjusted life years and costs associated with dengue.

We measured the effectiveness of each strategy in quality-adjusted life years (QALYs) to account for both time and quality of life. Specifically, we calculated the time-discounted QALYs lost to DF, DHF/DSS, and dengue-related deaths. A disability weight of one was used for premature death. The rate of new DF cases, DHF cases, and dengue-related deaths (Death_*k*_) in age group *k* was calculated as follows:

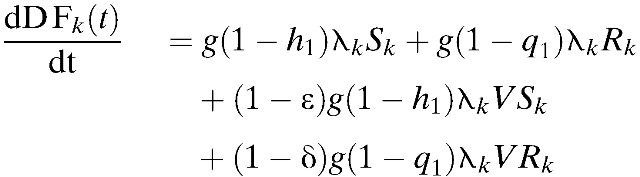


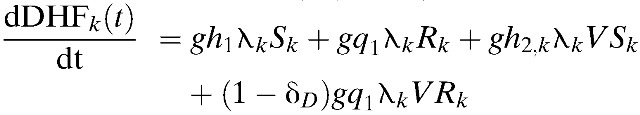


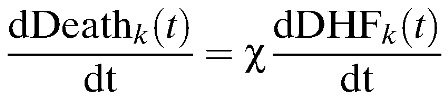


Using the equations above as well as the following equation, we calculated the number of dengue episodes and QALYs lost in each case^45^–^54^:

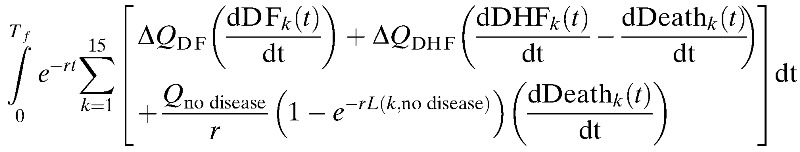

Here, *Q*_no disease_ is the quality of life in the absence of dengue infection (assumed to be one), *L*(*k*, no disease) is the residual expected lifespan of an individual in the age group *k* in the absence of dengue infection, *r* is the social discount rate of 3%, and *Q*_DF_ and *Q*_DHF_ are the quality of life lost per episode of DF and DHF (Table 2). To calculate the health effects, the quality-adjusted life expectancy (QALE) was first calculated in the case of a lethal dengue infection as:

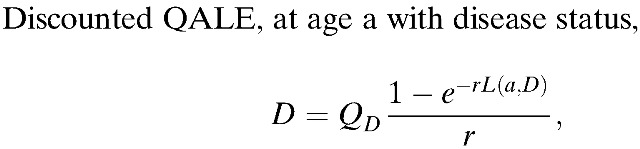

where *Q*_*D*_ is the quality of life associated with a disease state (*D*) and *L* is the residual life expectancy for an individual considering the life expectancy in the Philippines is 70 years.^53^,^55^ Therefore, the discounted QALY loss at age *a*, associated with dengue-related deaths, can be calculated as:

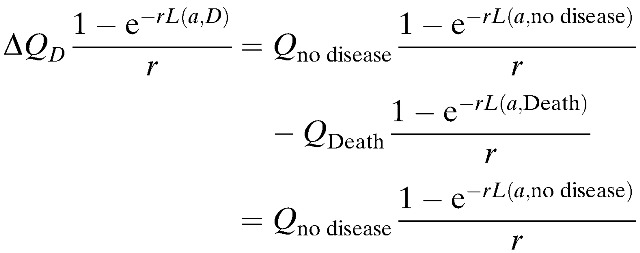

For the associated nonlethal infections, the QALY loss for DF and DHF is *Q*_DF_ and *Q*_DHF_, respectively.

In addition, the total costs accrued due to medical treatment, vaccination, and lost productivity is estimated by the following:

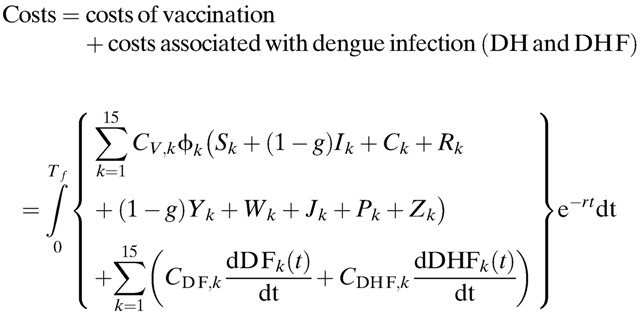

For the cost-effectiveness analysis from the health-care perspective, only direct costs were considered in the above equation, whereas both direct and indirect costs were considered from the societal perspective.

### Cost-effectiveness of dengue vaccination.

To analyze the cost-effectiveness of a vaccination program, we considered the balance between the cost of vaccination and the resulting incremental health effects. For our analysis, incremental effects were the differences between the incidence of dengue infection with and without the vaccination program. As customary in analyses of cost-effectiveness, the results are presented in units of cost per QALY gained by vaccination (compared with no vaccination) to express the cost of purchasing a year of good health. The discounted costs and benefits of a dengue vaccination program were summed over a time horizon of 20 years. To calculate the cost-effectiveness of the vaccine, we used the formula of the incremental cost-effectiveness ratio (ICER), that is, the cost per QALY gained by vaccination. The formula for ICER is as follows:

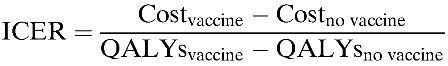

Consistent with the WHO criteria, vaccination is considered to be very cost-effective when ICER is less than the GDP per capita, is cost-effective when ICER is 1–3 times the GDP per capita, and is not effective when ICER exceeded three times the GDP per capita.^56^

## Results

### Disease burden of dengue in the Philippines.

**Figure 5.**
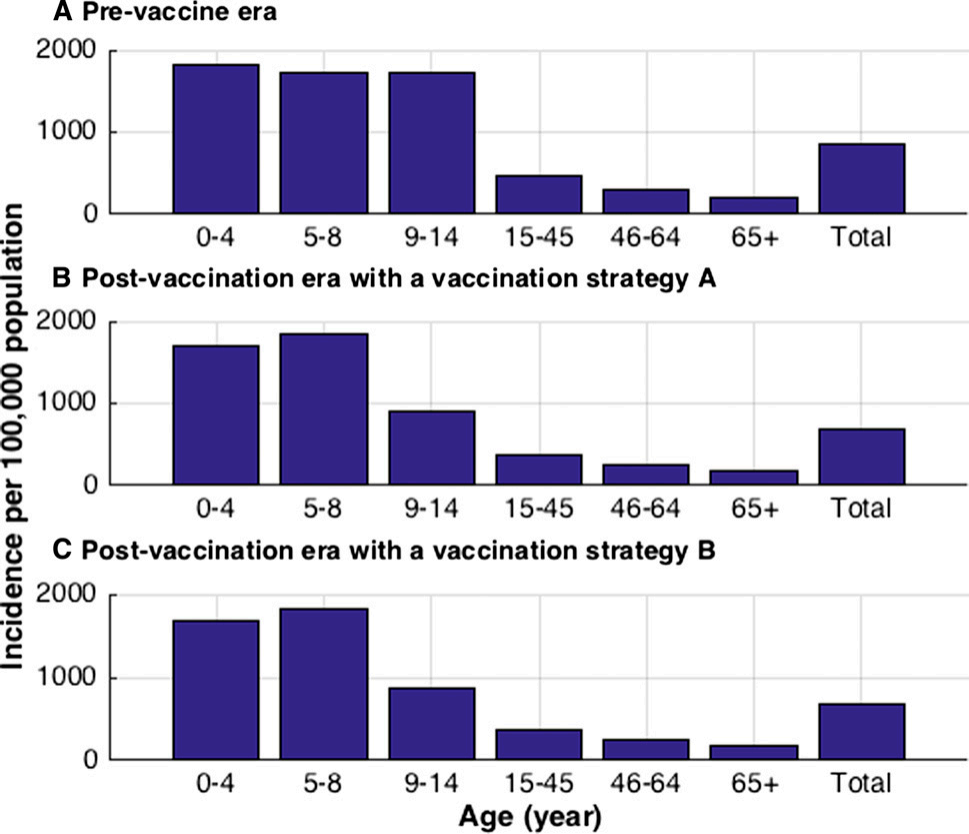
Expected annual number of symptomatic cases of dengue per 100,000 in respective age groups. (**A**) Expected annual incidence of symptomatic dengue in the prevaccine era. (**B**) Expected annual incidence of symptomatic dengue with vaccine Strategy A. (**C**) Expected annual incidence of symptomatic dengue with vaccine Strategy B.

We first calculated the annual dengue infection incidence in the absence of vaccination by simulating our model with our baseline parameter values. Our model was then fitted to the dengue incidence data presented in Figure 4, so that the estimated annual dengue infection incidence in the Philippines, including both asymptomatic and symptomatic infections, was 4.3%.^3^ Due to uncertainty in the asymptomatic rates in each age group, we multiplied the primary and secondary incidence for all age groups by a constant symptomatic rate of 23% (*g* = 0.23) to get symptomatic dengue incidence. On the basis of this assumption, the annual symptomatic dengue incidence was estimated to be 0.84%, which includes the annual DHF incidence of 0.016%. The expected annual number of symptomatic cases of dengue per age group in the prevaccine era is presented in Figure 5A

, which is comparable to the empirical data (Figure 4).^3^ In our simulation results, the highest incidence (1,720–1,810 per 100,000) occurred among individuals under 14 years of age, consistent with the observed pattern.^3^ Across all age groups, the annual incidence of symptomatic dengue per 100,000 individuals is estimated to be 860 cases in the prevaccine era.

### Epidemiological impact of dengue vaccination.

With vaccination strategies A and B, the average annual symptomatic incidence of dengue is expected to be 690 and 678 cases per 100,000 individuals, respectively (Figure 5). In addition, dengue vaccination affected the incidence of DHF. Specifically, above 20 years, vaccination strategies A and B reduced the incidence of DHF by 5% and 6% in all ages, respectively (Table 4). However, the incidence of DHF is expected to increase by 1% and 0.5% among 9-year-old individuals with vaccination strategies A and B, respectively. This is because vaccination of seronegative individuals potentially increases the risk of DHF during a subsequent wild-type infection. Furthermore, dengue vaccination had greater effects in the early stages of a vaccination program than later stages. Specifically, at 5 and 10 years after implementing vaccine Strategy A, the incidence of DF would be reduced by 13% and 17%, respectively, compared with prevaccine era. Similarly, at 5 and 10 years after adopting vaccine Strategy B, the incidence of DF would be reduced by 14% and 18%, respectively, and the incidence of DHF would be reduced by 5% and 7%, respectively. Lastly, our simulation results indicate that by year 20, the vaccination strategies A and B would reduce the incidence of DF and DHF by at least 20% and 9%, respectively, compared with prevaccine era.

### Vaccine cost-effectiveness.

From a health-care perspective, our model estimated that it would cost $7,687,887 U.S. dollars to treat dengue infections in a population of 100,000 individuals with no vaccination program. However, it would cost $6,615,860 or $6,521,900 to treat dengue infections if a vaccination program using Strategy A or B is implemented, respectively. From a societal perspective, Strategy A would reduce the cost of treating dengue infections in a population of 100,000 individuals from $8,494,586 to $7,332,538 and Strategy B would reduce the costs associated with treating dengue infections to $7,229,170.

**Figure 6.**
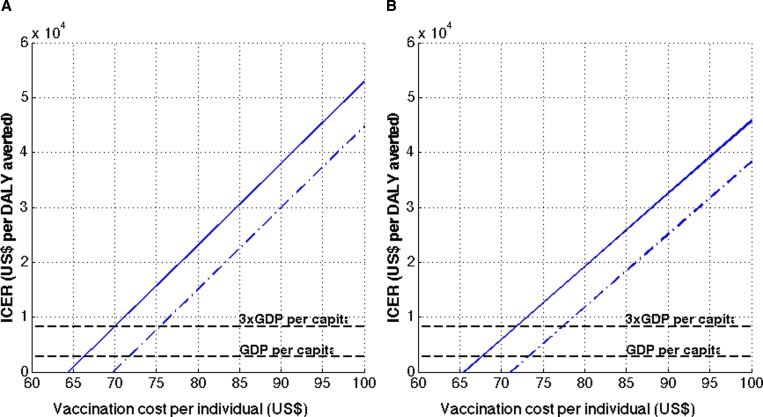
Cost-effectiveness of dengue vaccines. Cost-effectiveness ratios of dengue vaccination per quality-adjusted life year gained are presented (**A**) using Strategy A, and (**B**) using Strategy B. The solid lines indicate the cost-effectiveness ratios from health-care perspective, whereas the dashed lines indicate the cost-effectiveness ratios from societal perspective.

To calculate cost-effectiveness ratios, we considered a range of vaccine prices because the eventual price of the vaccine in the Philippines is currently uncertain. Therefore, instead of assuming that a single price would be determined, we estimated a threshold price below which a vaccination program would be (very) cost-effective. The cost threshold per person below which the vaccine started to be cost-effective increased from $70 for Strategy A to $72 for Strategy B, from a health-care perspective (Figure 6A

). Conservatively, the dengue vaccination program is “very cost-effective” from a health-care perspective when the cost of vaccination per person is under $66 and $68 with Strategy A and B, respectively. From a societal perspective, this cost threshold increases to $72 and to $74, respectively (Figure 6A and B). The threshold costs for a vaccine program to be cost-effective from a societal perspective increase to $75 with Strategy A and to $78 with Strategy B.

### Cost-effectiveness acceptability curve.

**Figure 7.**
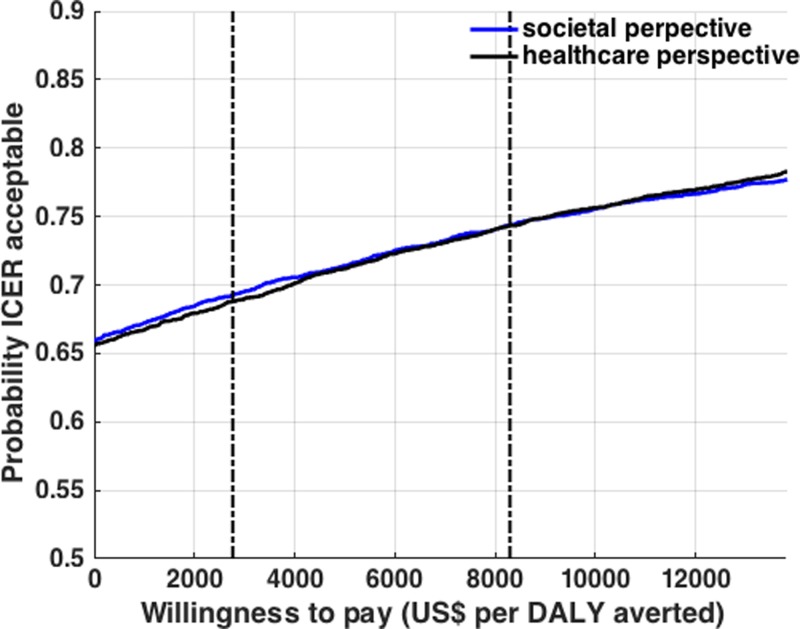
Cost-effectiveness acceptability curves. The curves show that dengue vaccination in the Philippines is cost-effective at different cost-effectiveness threshold values. The cost-effectiveness acceptability curves from health-care and societal perspectives are shown for assumed vaccine coverage levels shown with Strategy A, and if the cost of vaccination is fixed at $75 per individual.

To depict the likelihood that a chosen vaccination strategy is cost-effective over a range of acceptability thresholds, we carried out probabilistic sensitivity analysis by varying key parameters over distributions. Using this analysis with over 5,000 model iterations, we determined a cost-effectiveness acceptability curve. As an example, we chose the current vaccination program adopted in the Philippines shown in Strategy A, as well as a vaccination cost of $75 per individual (Figure 7

). This analysis revealed that from a health-care perspective, dengue vaccination is likely to be cost-effective at a willingness-to-pay value ≤ $2,765 (GDP per capita in the Philippines) per QALY in 69% of the model iterations. This likelihood of cost-effectiveness increased to 74% at an acceptability threshold of $8,295 (three times the GDP per capita in the Philippines) per QALY. From a societal perspective, the likelihood of cost-effectiveness is 68% and 74% at an acceptability threshold of $2,765 (GDP per capita) per QALY and $8,295 (three times the GDP per capita) per QALY, respectively.

## Discussion

Our analysis of the cost-effectiveness of the first dengue vaccination program with the CYD-TDV in the Philippines suggests that the vaccine would be cost-effective for a wide range of vaccine costs. Specifically, our model predicts that when 9-year-old individuals are consistently vaccinated (i.e., Strategy A) the vaccine will be cost-effective at costs as high as $75 from a societal perspective. These data are consistent with prior studies based on the data from the Americas.^45^,^50^–^58^

The fact that our findings are consistent with previous studies is meaningful because age patterns of dengue incidence are markedly different between SEA and the Americas. In the Americas, the predominant clinical expression of DF occurs in adults, whereas in SEA, severe dengue illnesses have been observed primarily in infants and children.^59^ On the basis of our results and those of prior studies, dengue vaccination has the potential to be cost-effective with carefully chosen target groups and sufficiently high vaccine uptake levels. Even with potential vaccine-induced increases in the risk of DHF, our study suggests that the dengue vaccine could remain cost-effective in the Philippines as long as the cost is less than $66 per person. Under the current vaccination regimen adopted in the Philippines (i.e., Strategy A), when the cost of vaccination is less than $64 per person vaccines incur a net savings per QALY. In other words, the avoided costs of treating dengue infection were greater than the costs of vaccination.

Nevertheless, our study is limited by several factors. First, there is uncertainty in existing dengue studies, mainly due to underreporting of symptomatic dengue infections and limited data on the type of treatment of episodes.^60^ In the Philippines, dengue surveillance depends mainly on disease reporting units and the current surveillance system focuses only on hospitalized cases. Thus, symptomatic dengue infections are underreported.^60^ Although we used an overall adjustment factor for dengue cases in the Philippines,^3^ future studies obtaining more accurate and comparable measures of the actual disease burden of dengue will greatly improve the estimates on the cost-effectiveness of dengue vaccination. Second, the mechanism of vaccine action in our model is only one possibility, whereas other possibilities have been mentioned in the literature, including age-dependent vaccine efficacy and waning vaccine immunity.^43^,^61^ Specifically, the cost-effectiveness of dengue vaccination would decrease when vaccine waning is considered. Third, our study did not account for broader impacts of dengue vaccination, such as reduced spending on outbreak control and averted losses in tourism.^62^ Although such factors were ignored in our analysis, we expect that incorporation of these broader benefits would result in greater economic value of dengue vaccination, thus improving its cost-effectiveness. In addition, although model inputs were drawn from an extensive review of the literature, the sources may vary in quality, and the model parameters may not hold under all conditions. Lastly, our model does not explicitly consider the vector biting process. Thus, our model combines the dynamics of infection in the vector and subsequent transmission to other humans into one aggregate rate, instead of considering separate contact rates for transmission from humans to vectors and vice versa. Yet many dengue studies, including evaluating the impact of vaccination, have been reasonably modeled without explicitly accounting for vector population dynamics.^16^,^17^ As a result, in existing mathematical models of dengue transmission, the vector population dynamics are often omitted,^14^,^15^,^18^–^28^ and rarely modeled explicitly.^16^,^17^ Nevertheless, for some modeling objectives including the evaluation of the impact of vector control efforts, inclusion of vector population dynamics would be helpful in providing more realistic model outcomes.

The goal of the global dengue strategy set forth by WHO aims to reduce dengue mortality by at least 50% by 2020, and to reduce dengue morbidity by at least 25% by 2020.^63^ However, the incidence of dengue is expected to increase due to various factors, including global warming, increases in population density, and the migration and international travel of infected people.^62^ Our analysis of the recently approved dengue vaccination program in the Philippines reveals that with appropriate vaccine pricing and uptake levels, dengue vaccination holds significant potential to confer excellent value and reduce the overall burden of dengue in the Philippines.

## Figures and Tables

**Table 1:** Epidemiological parameters

Symbol	Parameter	Value	Distribution	References
*f*_*k*_	Fertility rate in age group *k*	*f*_1_ = *f*_2_ = *f*_3_ = *f*_4_ = *f*_15_ = 0,*f*_5_ = *f*_6_ = *f*_7_ = *f*_8_ = *f*_9_ = *f*_10_ = 1.849 × 10^−4^,*f*_11_ = *f*_12_ = *f*_13_ = *f*_14_ = 2.740 × 10^−6^	Point estimate	29
*N*_*k*_	Relative size of age group *k*	*N*_1_ = 0.1168,*N*_2_ = 0.0858,*N*_3_ = 0.0215,*N*_4_ = 0.1073,*N*_5_ = *N*_6_ = *N*_7_ = *N*_8_ = *N*_9_ = *N*_10_ = 0.0777,*N*_11_ = *N*_12_ = *N*_13_ = *N*_14_ = 0.0398,*N*_15_ = 0.0432	Point estimate	29
*B*	Birth rate in Philippines, 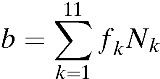 .	8.6690 × 10^−5^	Point estimate	–
*p*_*k*_	Rate of aging out of age group *k* (*p*_*k*_ = 1/*a*_*k*_, where *a*_*k*_ is the age interval in age group *k*)	*p*_3_ = 0.0027,*p*_*k*_ = 0.0005 for *k* ≠ 3	Point estimate	–
μ_*k*_	Death rate in age group *k*	μ_1_ = *b*/*N*_1_ − *p*_1_μ_*k*_ = *p*_*k* − 1_*N*_*k* − 1_/*N*_*k*_ − *p*_*k*_ (*k* ≠ 1)	Point estimate	–
β_*k*_	Transmission rate among age group *k*	β_1_ = 0.5121, β_2_ = 0.5536, β_3_ = 0.5536, β_4_ = 0.7058, β_5_ = β_6_ = β_7_ = β_8_ = β_9_ = β_10_ = 0.2768,β_11_ = β_12_ = β_13_ = β_14_ = 0.2007,β_15_ = 0.1522	Point estimate	Data fitting
σ_*n*_	Relative probability of being susceptible to *n*th infection	(5 − *n*)/4	Point estimate	30
ϕ_*k*_	Vaccination rate in age group *k*	φ_3_ = 0.00174 and φ_*k*_ = 0 for *k* ≠ 3 for Strategy Aφ_3_ = φ_4_ = 0.00174 and φ_*k*_ = 0 for *k* ≠ 3 or 4 for Strategy B	Point estimate	Author's assumption
ϵ	Vaccine efficacy against infection among the seronegative ≥ 9 years of age	0.616	Point estimate	31
δ	Vaccine efficacy against infection among the seropositive ≥ 9 years of age	0.792	Point estimate	31
δ_*D*_	Vaccine efficacy against DHF among the seropositive ≥ 9 years of age	0.909	Point estimate	31
*g*	Proportion of dengue infections that are symptomatic	0.23	Beta (7, 23)	32,33
γ	Rate of recovery from infection	0.146/day	Point estimate	15
γ_*C*_	Rate of loss of cross-immunity	0.0055/day	Beta (37.3, 6,790)	16,34
*h*_1_	Probability of developing DHF/DSS after primary symptomatic infection among the unvaccinated	0.00245	Beta (5, 2,037)	13,14,30
*h*_2*,k*_	Probability of developing DHF/DSS after primary symptomatic infection among those vaccinated who were seronegative when vaccinated	0.114 for *k* = 1, 2	Point estimate	9
		0.048 for k = 3, …, 15		
*q*_1_	Probability of developing DHF/DSS after secondary symptomatic infection among the unvaccinated	0.0448	Beta (50, 1,066)	13,14,30
χ	Risk of death from DHF/DSS	0.01	Beta (2,198)	30,35

DHF = dengue hemorrhagic fever; DSS = dengue shock syndrome. Parameter values were used in the analysis unless indicated otherwise.

**Table 2:** Cost-effectiveness parameters

Symbol	Parameter	Value	Distribution	References
*r*	Social discount rate for QALYs calculations	0.03	Point estimate	45,46
*D*_Death_	Disability weight for death	1	Point estimate	45,46
*D*_DF_	Disability weight for DF	0.197	Beta (19.7, 80.3)	30,47
*D*_DHF_	Disability weight for DHF/DSS	0.545	Beta (54.5, 45.5)	30,47
*L*_DF_	Time lost due to DF (years)	0.019	Beta (5.7, 294.3)	43,30,48,49
*L*_DHF_	Time lost due to DHF/DSS (years)	0.0325	Beta (13, 387)	43,30
*L*_Death,*k*_	Years of life lost due to death for age group *k*	67.5 for *k* = 1,63 for *k* = 2,61 for *k* = 3,57.5 − 5 (*k* − 4) for *k* = 4, …, 15	Point estimate	
*a*_*k*_	Average age of dengue exposure in age class *k*	2.5 for *k* = 1,7 for *k* = 2,9 for *k* = 3,5 (*k* − 4) + 12.5 for *k* = 4, …, 15	Point estimate	

DF = dengue fever; DHF = dengue hemorrhagic fever; DSS = dengue shock syndrome; QALY = quality-adjusted life year.

**Table 3:** Probabilities and costs of dengue infection

	Probability	Relative probability	Direct medical costs ($)	Indirect costs ($)	References
Any primary dengue infection	1.00				
Asymptomatic	0.75 (= 1 − *g*)	1.00	135	20	32
Symptomatic	0.25 (= *g*)	0.9976 (= 1 − κ_1_)	636	42	32
DF		0.3591 = 0.36 (1 − κ_1_)	636	42	13,14,30
Ambulatory		0.6385 = 0.64 (1 − κ_1_)	NA	87,418 for children (< 15 years of age) 56,822 for adults (≥ 15 years of age)	2,3,50
Hospitalized		0.0024 (= κ_1_)			2,3,50
Severe (DHF)		0.00243 = (1 − χ) κ_1_			13,14,30
Hospitalized		2.45 × 10^−5^ = χ κ_1_			2,3,30,35
Death					30,35,51,52
Any secondary dengue infection	1.00				
Asymptomatic	0.75 (= 1 − *g*)	1.00	135	20	32
Symptomatic	0.25 (= *g*)	0.9552 (= 1 − κ_2_)	636	42	32
DF		0.3439 = 0.36 (1 − κ_2_)	636	42	13,14,30
Ambulatory		0.6113 = 0.64 (1 − κ_2_)	NA	197,622	2,3,50
Hospitalized		0.0448 (= κ_2_)			2,3,50
Severe (DHF)		0.0444 = (1 − χ) κ_1_			13,14,30
Hospitalized		4.48 × 10^−4^ = χ κ_1_			2,3,30,35
Death					30,35,51,52

DF = dengue fever; DHF = dengue hemorrhagic fever; NA = not applicable. All values are reported in 2016 U.S. dollars.

**Table 4:** Annual dengue cases with and without a vaccination program

	Prevaccine era	Vaccination Strategy C	Vaccination Strategy B	Vaccination Strategy A
Symptomatic infection (%)	0.91	0.73	0.51	0.18
Primary infection (%)	0.52	0.45	0.34	0.14
Secondary infection (%)	0.39	0.28	0.17	0.04
No. of DHF cases per million	187	140	83	22

DHF = dengue hemorrhagic fever.
